# Economic Evaluations of Public Health Interventions to Improve Mental Health and Prevent Suicidal Thoughts and Behaviours: A Systematic Literature Review

**DOI:** 10.1007/s10488-020-01072-9

**Published:** 2020-07-30

**Authors:** Inna Feldman, Mihretab Gebreslassie, Filipa Sampaio, Camilla Nystrand, Richard Ssegonja

**Affiliations:** Department of Public Health and Caring Sciences, Uppsala University, Husargatan 3, P.O Box 564, 751 22 Uppsala, Sweden

**Keywords:** Economic evaluation, Systematic review, Quality assessment, Mental health, Suicide

## Abstract

To review the literature on economic evaluations of public health interventions targeting prevention of mental health problems and suicide, to support evidence based societal resource allocation. A systematic review of economic evaluations within mental health and suicide prevention was conducted including studies published between January 2000 and November 2018. The studies were identified through Medline, PsychINFO, Web of Science, the National Health Service Economic Evaluation Database and Health Technology Assessment. The quality of relevant studies and the transferability of their results were assessed using a criterion set out by the Swedish Agency for Health Technology Assessment. Nineteen studies of moderate to high quality were included in this review, which evaluated 18 interventions in mental health and four interventions in suicide prevention. Fourteen (63%) of all interventions were cost-effective based on the conclusions from original papers. None of the studies that evaluated suicide prevention was of high quality. The interventions largely focused on psychological interventions at school, the workplace and within elderly care as well as screening and brief interventions in primary care. Nine studies (around 50% of included articles) had a high potential for transferability to the Swedish context. Public health interventions aiming to improve mental health have a high potential to be economically beneficial to society, but high-quality evidence on the cost-effectiveness of suicide prevention is limited.

**Electronic supplementary material:**

The online version of this article (10.1007/s10488-020-01072-9) contains supplementary material, which is available to authorized users.

## Introduction

Mental health problems account for a substantial burden of disease globally, with the World Health Organization predicting that by 2030, mental health problems will be the highest ranking disease area in terms of burden in affluent countries (Mathers and Loncar 2006). Therefore, prevention of mental health problems has received considerable attention in integrated mental health policies in nearly all countries in the European Region. The economic burden of mental health problems is substantial (DiLuca and Olesen 2014; McDaid et al. 2010). Specifically, mental health problems arising in childhood and adolescence are burdensome, since they may lead to high societal costs throughout life if not treated. A recently published longitudinal cost-of illness study (Ssegonja et al. 2019) confirmed that depression in adolescence is associated with increased healthcare consumption in mid-adulthood, estimating an additional annual cost of 3.1 million USD for a single age cohort of females with a history of persistent depressive disorder. Similarly, conduct problems in childhood increase the risk of adverse outcomes such as school failure, unemployment, antisocial and criminal behaviour, and alcohol and drug abuse (Fergusson et al. 2005; Knapp et al. 2011). Collectively, mental health problems place a high financial burden on the individual, families and society (Romeo et al. 2006; Scott et al. 2001).

Increasing the attention towards mental health promotion within the public health arena could help avoid some of the economic burden of poor mental health and could potentially be economically attractive investments (Mihalopoulos et al. 2011a, b). Context relevant strategies spanning the broad spectrum of prevention, including both population-based interventions and interventions targeting high-risk individuals, are called for to reduce the burden associated with mental health problems. When focusing on population level interventions, even small improvements can translate into significant public health gains (Sarkadi et al. 2014). As any public health approach, mental health interventions intend to promote or protect mental health or prevent mental ill health in communities or populations. They can be distinguished from clinical interventions, which are intended to prevent or treat mental illness in individuals, and tend to be complex, pragmatic, and context dependent (Rychetnik et al. 2002). Studies on mental ill health and its associated economic burden draw attention to the substantial consequences of mental health problems. However, they cannot answer the question of whether investing resources into preventing such problems is an economically sound use of scarce resources compared to their alternative use or application. For this purpose, evidence from economic evaluations should be applied, whereby benefits and costs of alternative interventions are considered to aid decision-makers in prioritizing and allocating resources (Drummond et al. 2005). More recently, arguments have also been put forward to examine the economic case of all areas of public health and health promotion (Gallagher 2005), and while some studies have been conducted in these areas, such evaluations remain scarce compared with healthcare interventions (McDaid and Needle 2009).

At the same time, in the context of budget constraints, economic evaluations can provide important information to aid public health decision-making, including (1) shedding light on which interventions that are effective and in what context and (2) information on the economic costs and consequences of such interventions. However, the use of evidence from economic evaluations is limited for the majority of countries. A number of reasons may account for this, such as difficulties associated with applying economic evidence in different settings, and in which settings interventions aimed to improve mental health are effective. Despite the above observation, the use of economic evaluations in the field of mental health care generally continues to grow (Evers et al. 2007). Some systematic reviews summarizing the economic impact of interventions designed to prevent mental disorders were recently published (Camacho and Shields 2018; Mihalopoulos and Chatterton 2015; Paganini et al. 2018). The majority of the interventions included were implemented in the health care sector and targeted distinct diagnoses, such as depression and anxiety. Economic evaluations of worksite interventions aiming to prevent or treat mental health problems were summarized in another systematic review (Hamberg-van Reenen et al. 2012). The general conclusion was that interventions were either lacking methodological quality or effectiveness evidence, thus only a tentative conclusion could be drawn from the study results. Another review published in (Zechmeister et al. 2008) summarized the economic literature on the prevention of mental health disorders and promotion of mental well-being. The authors concluded that evidence on cost-effectiveness was limited to a very small number of interventions with restricted scope for generalizability and transferability. That is why the recent work by (Zechmeister et al. 2008) recommends that prioritization of different interventions should use more evidence from country-and population-specific economic evaluations.

To our understanding, current evidence on the cost-effectiveness of different interventions that focus solely on prevention of mental health problems in different settings is limited. Thus, the aims of this study were: (1) to review the current literature on economic evaluations of public health interventions targeting the prevention of mental health problems and suicidal thoughts and actions, (2) to assess the transferability of the results to the Swedish context. The study was a part of a larger project conducted on behalf of The Public Health Agency of Sweden, with the overall aim of summarizing the results from existing economic evaluations of different interventions targeting central areas of public health, such as physical activity and diet, alcohol, narcotics, doping, tobacco, and gambling (ANDTG), mental health and suicide. This systematic literature review was registered in PROSPERO database as “Economic evaluations of public health interventions to improve mental health and prevent suicidal thoughts and actions: A systematic literature review”, reg. number: CRD42018117634.

## Methods

### Eligibility Criteria

This review followed the PRISMA (Preferred Reporting Items for Systematic Reviews and Meta-Analyses) guidelines statement 2009 (Moher et al. 2009) and other recommended guidelines for systematic reviews (Khan et al. 2003; van Mastrigt et al. 2016). The overall search was guided by structured inclusion and exclusion criteria in PICOS format (Khan et al. 2003) (see Table 1).

**Table 1:** Search structure according to PICOS-components with inclusion and exclusion criteria

PICOS-component	Inclusion criteria	Exclusion criteria
Population	All age groups, the total population (healthy) or some population group that already have or are at a risk of experiencing mental health problems/suicidal thoughts/behaviour	People who have already developed a mental health disorder
Intervention	All types of universal and indicated measures, delivered outside of hospitals i.e., programs implemented in schools, elderly care, through social services, at workplaces, within primary care or through voluntary organizations	Selective prevention measures and treatments for already sick individuals with a mental health disorder
Comparator	Any active or passive comparator	No comparator
Outcomes	Cost per QALY and/or cost per DALY	If the study reports costs in relation to only clinical outcomes
Study design	Full economic evaluations of any type (empirical and model based) that include both cost and health outcomes	Partial economic evaluations that report only outcomes/costs, or that only include intervention costs (no cost consequences). Economic evaluations based on clinical studies without follow-up. Qualitative studies and study protocols

*QALY* quality adjusted life years, *DALY* disability adjusted life years;

Each intervention was classified using the mental health intervention spectrum developed by Mrazek and Haggerty (1994). A preventive intervention was considered universal if it targeted a whole population. Examples include prenatal care and immunizations. Selective preventative interventions targeted a subgroup of the population at risk of developing a mental disorder or problems (e.g. children of mothers who had depression). Indicated preventive interventions targeted high-risk individuals who had symptoms of a mental disorder but did not meet the diagnostic criteria. In this review, we included universal and indicated interventions only due to their potential for generalizability in comparison to selective interventions. Additionally, we only included studies that measured health outcomes using a generic metric combining both time and quality of life (i.e. mortality and morbidity effects) using preference-based techniques. The most widely used generic preference-based outcome measure is the quality adjusted life year (QALY) and the disability-adjusted life-year (DALY). QALYs and DALYs are determined by multiplying the length of time in a particular health state by a weight that denotes the quality of life or disability associated with that health state. The weights are defined on a scale of 0–1, where for QALYs 0 denotes death and 1 denotes perfect health. For DALYs, the 0–1 scale is inverted. Descriptive economic studies, which did not include both costs and benefits of interventions, were excluded, as well as systematic reviews, comments and letters to editors, conference abstracts and studies without access to the full text. Furthermore, we excluded studies conducted outside Europe, USA, Canada, Australia and New Zeeland, as well as articles published in languages other than English or Swedish.

### Information Sources and Search Strategy

A search was undertaken in a range of bibliographic databases, including PubMed, Web of Science, PsycINFO, National Health Service Economic Evaluation Databases (NHS EED) and the Health Technology Assessment Database (HTA). The search was done between 21 and 27th of November 2018 and was limited to articles published from year 2000 onwards. This was further complemented by a search for grey literature, including governmental reports and academic working papers. The primary search was based on defined search words and search terms, Medical Subject Headings (MeSH). We used the InterTASC Information Specialists' Sub-Group website (InterTASC) as well as other published systematic reviews (Hamberg-van Reenen et al. 2012; Mihalopoulos and Chatterton 2015; Mihalopoulos et al. 2011a, b; Zechmeister et al. 2008) to look for relevant search terms and filters. Precise search terms included core medical subject headings and title/abstract phrases including 'mental disorder', 'mental illness', 'stress', 'mental wellbeing', ‘(primary) prevention', 'health promotion', 'prevention' and 'suicide'. In addition to the use of the MeSH terms 'costs' and ‘cost analysis', other economic terms such as 'economics', 'cost effectiveness', 'cost utility', and 'economic evaluation' were used. Grey literature was identified through a secondary search in websites containing guidelines and recommendations for interventions within the relevant areas; the Swedish National Board of Health and Welfare, the Public Health Agency of Sweden and the Swedish Institute for Health Economics. The search strategy is presented in detail in Online Appendix 1.

To validate our search strategy, we scanned references in the identified systematic reviews (Clarke et al. 2015; Mihalopoulos and Chatterton 2015; Mihalopoulos et al. 2011a, b; Zechmeister et al. 2008) from our primary search. Whereas the majority of the references were already included in the primary search results, the remaining studies were assessed using the inclusion and exclusion criteria and were rated as irrelevant. Therefore, we assumed that our search strategy was broad enough to find the appropriate studies.

### Relevance and Quality Assessment

Two independent reviewers screened all retrieved articles for title and abstract relevance according to the PICOS criteria. To assess agreement between author assessments, a random 20% of the selected abstracts were additionally screened independently by one of the authors. An agreement statistic (Cohen's Kappa) was calculated, ranging between 0.59 and 1.00, representing good agreement (Orwin and Vevea 2009). The selected articles were read in full text, and study quality was assessed using checklists created by the Swedish Health Technology Agency ("Appendix 7. Checklist for assessing the quality of trialbased health economic studies" 2018a; "Appendix 8. Checklist for assessing the quality of of health economic modelling studies" 2018b), which are similar to commonly used guidelines and reporting checklists (Husereau et al. 2013). The checklists included four areas for reporting: (1) study relevance, (2) transferability to the Swedish context, (3) conflict of interest, and (4) study quality. Assessment of transferability (2) included the relevance of the intervention, delivery mode and setting and relevance of unit costs to Swedish context. The checklists included the following individual items: costs analysis, measurement of effectiveness and valuation of preference-based outcomes, evaluation method/structure, parameters included in the analysis, and interpretation of results, discounting and uncertainty. Every area included a range of questions with alternative answers: yes/no/unclear/not applicable.

The checklist results were summarized according to three categories: (a) transferability (b) methodological quality of the study regarding economic aspects and (c) the effects and side effects of the intervention. For each category, the studies could be classified as ‘High', 'Moderate', 'Low' or 'Insufficient' quality. An exclusion condition for lack of quality was also devised. If the study had 'Low' or 'Insufficient' quality in any of the three categories, it was judged to be of poor quality, and was thus excluded from the review. A study was considered as 'High' quality if it was classified with 'High' quality in all three categories. The checklist was completed for each study included in the review by one author (MG), and 20% of the articles were later reviewed by the other authors (IF, FS, CN, RS). Agreement between the authors was 0.69.

### Data Extraction

The results of the review were synthesized in a narrative and tabular format. This included information about author and country, target population and setting, intervention and comparator, type of economic evaluation, costing perspective, time horizon and discount rate, outcome measurements, costs included, and results presented as an incremental cost-effectiveness ratio (ICER). Each intervention was classified as either universal or indicated within the target areas (mental health or suicide). Data were extracted by one of the authors, and later 20% of the articles were randomly assigned to the other authors for data extraction, which resulted in 83% agreement. Results were reported per intervention, rather than per study, as many studies evaluated several interventions.

Generally, an intervention is cost effective if there is a high probability (more than 50%) that the incremental cost-effectiveness ratio (ICER) is below a given willingness to pay (WTP) threshold compared to the alternative course of action. A cost-effectiveness threshold is generally set so that the interventions that appear to be relatively good or very good value for money can be identified. The most commonly cited cost-effectiveness thresholds are between one and three per-capita gross domestic product (GDP) (Hutubessy et al. 2003). For the majority of the included countries, WTP was between EUR 50,000 and EUR 80,000. An intervention was considered cost-effective in this review, if authors of the original paper explicitly stated such.

## Results

### Search Outcomes and Overview of the Included Studies

**Fig. 1:**
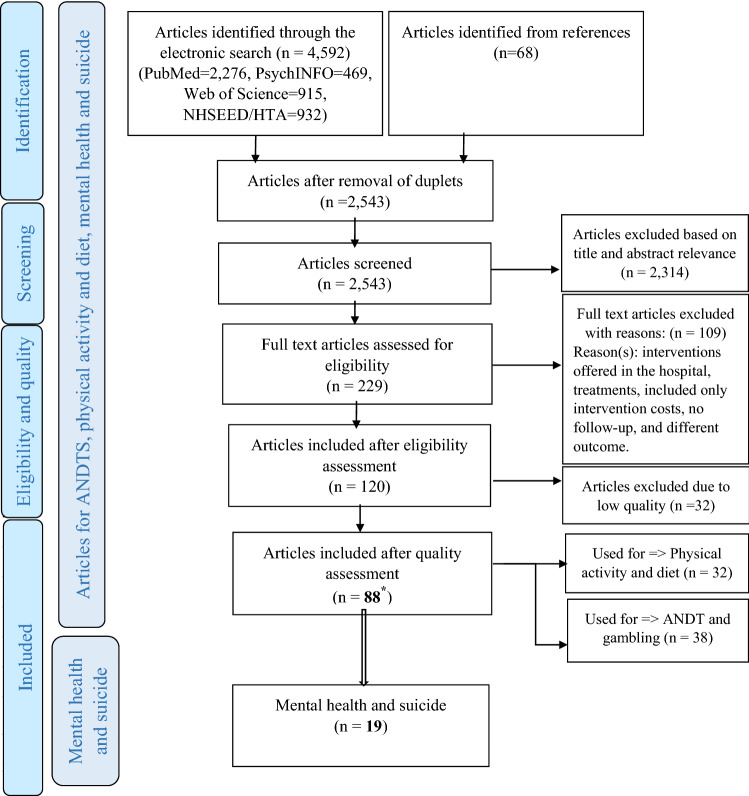
Flow diagram of the included articles according to PRISMA. ^*^The total number of articles in the different categories does not add up to 88 articles because one article reports results of interventions targeting more than one category

The search process resulted in 2543 hits for all public health areas, including mental health and suicide, after removing duplicates. No relevant studies were found in the grey literature. Full texts were reviewed for 229 of the articles, and 109 of these were excluded due to irrelevance, and a further 32 were removed due to ‘Low’ or ‘Insufficient’ quality. Full information regarding the review findings and screening process is shown in Fig. 1 (PRISMA diagram). The diagram illustrates the full review process including mental health and suicide as well as ANDTG, physical activity and diet. Nineteen studies were included in this review, containing 22 different interventions.

Details on all studies with “High” and “Moderate” quality can be found in Table 2.

**Table 2:** Included studies, interventions, quality and cost-effectiveness

Target area	Number of included interventions		Number of included studies	Number of studies with high quality	Cost-effective interventions (according to authors’)
Mental Health (MH)	Mental Health (MH)	8	8	0	4
	Indicated	10	10	4	8
Suicide	Universal	1	1	0	1
	Indicated	3	2	0	1
Total MH and Suicide		22	19	4	14

The majority of these studies evaluated interventions in European countries (United Kingdom, the Netherlands, Germany, Spain and Sweden) with the remainder conducted in Australia (4) and USA (1). Seventeen studies (18 interventions) targeting mental health were included and two studies (four interventions) targeting suicide.

Most of the studies (14) were empirical evaluations based on data from randomized control trials (RCT) with 6–24 months follow-up periods, and only five of the 19 studies (Comans et al. 2013; Lee et al. 2017; Mihalopoulos et al. 2012; Ophuis et al. 2018; van den Berg et al. 2011) were model-based evaluations. Almost half of the interventions were universal, targeting the whole population regardless of the level of underlying problems or risk factors. A summary of the type of study/intervention, the proportion of studies with high quality, and the proportion of cost-effective results are presented in Table 3 and the detailed results for every study/intervention are presented in Tables 4 and 5.

**Table 3:** Quality and transferability assessment of studies rated as “moderate” or “high” quality

N	Author/year	Field	Transferability of the study's economic results	Study quality with respect to economic aspects	Study quality with respect to the effects and side effects of the intervention	Overall
1	Anderson et al. (2014)	Mental health	High	high	High	High
2	Bosmans et al. (2014)	Mental health	High	Moderate	Moderate	Moderate
3	Buntrock et al. (2017)	Mental health	High	High	High	High
4	Coulton et al. (2015)	Mental health	Moderate	Moderate	Moderate	Moderate
5	Fernández et al. (2018)	Mental health	Moderate	High	High	Moderate
6	Lee et al. (2017)	Mental health	Moderate	Moderate	Moderate	Moderate
7	Lewis et al. (2017)	Mental health	High	High	High	High
8	Lynch et al. (2005)	Mental health	Moderate	High	High	Moderate
9	Mihalopoulos et al. (2012)	Mental health	Moderate	Moderate	High	Moderate
10	Oostrom et al. (2010)	Mental health	High	High	High	High
11	Ophuis et al. (2018)	Mental health	Moderate	Moderate	High	Moderate
12	Philipsson et al. (2013)	Mental health	High	High	Moderate	Moderate
13	Ride et al. (2016)	Mental health	Moderate	High	High	Moderate
14	Stallard et al. (2015)	Mental health	Moderate	Moderate	Moderate	Moderate
15	Uegaki et al. (2011)	Mental health	High	Moderate	Moderate	Moderate
16	Underwood et al. (2013)	Mental health	Moderate	Moderate	Moderate	Moderate
17	van den Berg et al. (2011)	Mental health	High	Moderate	Moderate	Moderate
18	Ahern et al. (2018)	Suicide	High	Moderate	Moderate	Moderate
19	Comans et al. (2013)	Suicide	Moderate	Moderate	Moderate	Moderate

**Table 4:** Description of the included studies/interventions

N	Author/year/country	Population/setting	Intervention(s)	Intervention type
1	Anderson et al. (2014), UK	Children aged 12–16 years/School	1. Classroom-based CBT (The Resourceful Adolescent Programme (RAP)) 2. Usual school provision of Personal Social and Health Education	Universal
2	Bosmans et al. (2014), Netherlands	General population/Elderly	1. Stepped care prevention programme 2. Usual care	Indicated
3	Buntrock et al. (2017), Germany	Adults (≥ 18 years) with subsyndromal depression/Community	1. Web-based guided self-help 2. Enhanced usual care (usual care supplemented with Web-based psycho-education)	Indicated
4	Coulton et al. (2015), UK	Adults (≥ 60 years)/Community)	1. Community group singing 2. Usual care	Universal
5	Fernández et al. (2018), Spain	Adults (18–75 years)/Primary care centres	1. The use of PredictD risk algorithm 2. Usual care	Indicated
6	Lee et al. (2017), Australia	Adolescents (11–17 years)/School	1. Universal School-based psychological interventions 2. No intervention	Univerl
		Adolescents (11–17 years)/School	1. Indicated School-based psychological interventions 2. No intervention	Indicated
7	Lewis (2017), UK	Elders with subsyndromal depression based /Primary care centres	1. Collaborative care and active surveillance 2. Usual care	Indicated
8	Lynch et al. (2005), US	Teenagers (13–18 years) at risk of or with subsyndromal depression /Health maintenance organization (HMO)	1. Cognitive behavioural intervention 2. Usual care	Indicated
9	Mihalopoulos et al. (2012), Australia	Children (11–17 years) screened for depression symptoms/School	1. Psychological intervention 2. No intervention	Indicated
10	Oostrom et al. (2010), Netherlands	Sick-listed employees with distress (for 2–8 weeks)/Work place	1. Workplace intervention 2. Usual care	Indicated
11	Ophuis et al. (2018), Netherlands	Adult population aged 18 to 65 years, with subthreshold panic disorder /Primary care centres	1. CBT-based early intervention 2. Usual care	Indicated
12	Philipsson et al. (2013), Sweden	Adolescent girls (13–18 years) with internalizing problems/School	1. Dance intervention 2. Usual care	Indicated
13	Ride et al. (2016), Australia	First time mothers/Maternal and Child Health Centres	1. What Were We Thinking (WWWT) 2. Usual care	Universal
14	Stallard et al. (2015), UK	Children (9–10 years)/School	1. FRIENDS programme 2. Usual care and school-led FRIENDS	Universal
15	Uegaki et al. (2011), Netherlands	Working women of childbearing age during maternity leave/Homes	1. Supervisor telephone contact (STC) 2. Usual care	Universal
16	Underwood et al. (2013), UK	Adults (≥ 65 years)/Elderly homes)	1. OPERA (‘whole-home’ exercise) intervention 2. Current best care (an awareness program on depression)	Universal
17	van den Berg (2011), Netherlands	Adults (20–65 years) screened for subsyndromal depression/Primary care centres	1. Opportunistic screening and minimal contact psychotherapy 2. Usual care	Universal
18	Ahern et al. (2018), Europe	Students from 168 schools in ten European Union (EU) countries/School	1. Question, Persuade, Refer (QPR) 2. Control (posters with minor components of YAM)	Indicated
			1. Youth Aware of Mental Health programme (YAM) 2. Control (posters with minor components of YAM)	Universal
			1. Screening by Professionals (ProfScreen) 2. Control (posters with minor components of YAM)	Indicated
19	Comans et al. (2013), Australia	Adults (45 (SD = 15) years) bereaved by suicide/Community	1. StandBy Response Service 2. Usual care	Indicated

**Table 5:** Methods and summary of the findings of the included studies/interventions

N	Author/year/country	Study design/type	Perspective	discounting/time horizon	Costs data	Outcome(s)	Summary of findings
1	Anderson et al. (2014), UK	Trial based cost utility analysis	Societal	NA/NA	Intervention and healthcare costs; 2010 GBP	Symptoms of depression; QALY	ICER: GBP 747.37/SMFQ Dominated* (as measured in QALY). Authors' conclusion: not cost-effective
2	Bosmans et al. (2014), Netherlands	Trial based cost utility analysis	Societal	NA/NA	Direct costs (intervention and healthcare costs) and other societal costs: 2008 EUR	Incidence depression or anxiety; Incidence depression; Incidence anxiety; Improvement CES-D; Improvement HADS-A; QALY	ICERS: EUR 85,521/incidence of depression or anxiety; EUR 10,293/incidence of depression; EUR 10,328/incidence of anxiety; EUR 364/improvement in CES-D score; EUR 963/improvement in HADS-A score; EUR 26,890/QALYs and probability of CE ranged from 13 to 85% if WTP is 0 to infinity. Authors' conclusion: not cost-effective
3	Buntrock et al. (2017), Germany	Trial based cost utility analysis	Societal	NA/1 year	Intervention, healthcare, patient and family costs and productivity losses: 2013 EUR	Depression-free years (DFY); QALY	From societal perspective; EUR 1117/DFYs & EUR 13,400/QALYs, 99% probability of CE at a WTP of EUR 20,000/DFY & 60% at a WTP of EUR 20,000/QALY. From healthcare perspective; EUR 1125/DFYs, EUR 13,500/QALYs, 64% probability of CE at EUR 20,000/QALY WTP. Authors' conclusion: cost-effective
4	Coulton et al. (2015), UK	Trial based cost utility analysis	Societal	NA/NA	Intervention and health care costs (and some indirect costs like advertising and management costs): Unclear year, GBP	QALY	Incremental cost of GBP 24,75 and a gain of 0.015 QALYs, with 64% probability of CE at a WTP of GBP 30,000/QALY Authors' conclusion: cost-effective
5	Fernández et al. (2018), Spain	Trial based cost utility analysis	Societal	3.5%/NA	Direct costs (intervention and healthcare) and productivity loss: 2012 EUR	QALY	Main Outcome: (cost per QALY gained using EQ-5D) Societal perspective: Dominant** Healthcare sector perspective: an ICER of EUR 1327/QALY Secondary outcomes: (cost per QALY gained measured with VAS) Societal perspective: Dominant Healthcare sector perspective: an ICER of EUR 1085/QALY. Authors' conclusion: cost-effective
6	Lee et al. (2017), Australia	Model based cost utility analysis	Health and education sector	3.0%/10 years	Intervention and disease cost (offsets): 2013 AUD	DALY	AUD 7350/DALY and high probability of cost-effectiveness at a WTP of AUD 50,000/DALY. Authors' conclusion: cost-effective
							AUD 19,550/DALY and high probability of cost-effectiveness at a WTP of AUD 50,000/DALY. Authors' conclusion: cost-effective
7	Lewis et al. (2017), UK	Trial based cost utility analysis	Societal	NA/2 years	Intervention and health-care cost: 2012/13 GBP	QALY	An ICER of GBP 9633/QALY with 92% probability CE at a WTP of GBP 20,000 per QALY and 97% at a WTP of GBP 30,000 per QALY. Authors' conclusion: cost-effective
8	Lynch et al. (2005), US	Trial based cost utility analysis	Societal	NA/1 year	Intervention and health-care cost: 2000 USD	Depression-free days (DFDs); QALY	Estimated as cost/DFD: USD 10/DFD (95% CI − 13 to 52) Estimated as cost/QALY: USD 9275/QALY (95% CI − 12,148 to 45,641). Authors' conclusion: cost-effective
9	Mihalopoulos et al. (2012), Australia	Model based cost utility analysis	Healthcare	3.0%/6 years	Intervention and disease cost (offsets): 2003 AUD	DALY	ICER: AUD 5400 per DALY. Authors' conclusion: cost-effective
10	Oostrom (2010), Netherlands	Trial based cost utility analysis	Societal	NA/NA	Intervention and all healthcare-related costs and productivity loss: 2008 EUR	Lasting RTW; QALY	EUR 627/1-day reduction in sick leave, < 50% probability of CE regardless of the amount of WTP and it was dominated as measured in cost/QALY. Authors' conclusion: not cost-effective
11	Ophuis et al. (2018), Netherlands	Model based cost utility analysis	Societal	4.0% for costs and 1.5% for effects/5 years	Intervention, direct and indirect costs: 2014 EUR	QALY	Dominant with 98% probability of CE at a WTP of EUR 20,000 per QALY. Authors' conclusion: cost-effective
12	Philipsson et al. (2013), Sweden	Trial based cost utility analysis	Societal	3.0%/NA	Intervention (including overhead costs) and healthcare costs: 2011 USD	QALY	USD 3830/QALY with 95% probability of CE at a WTP of USD 50,000/QALY. Authors' conclusion: cost-effective
13	Ride et al. (2016), Australia	Trial based cost utility analysis	Public	NA/6 months	Public sector and participant out-of-pocket costs: 2013–2014 AUD	30-day prevalence of depression, anxiety and adjustment disorders: QALY	Cost per percentage-point reduction in 30-day prevalence of depression, anxiety and adjustment disorders was AUD 152 (95% CI − 16,453 to 16,756). Cost per QALY was AUD 36,451 (95% CI − 1,554,006 to 1,626,908). There was a 55% probability of CE at a WTP of AUD 55,000/QALY. Authors' conclusion: cost-effective
14	Stallard et al. (2015), UK	Trial based cost utility analysis	Healthcare and education (social services) sector	NA/NA	Intervention and healthcare cost: 2013 GBP	RCADS (Revised Child Anxiety and Depression Scale) score; QALY	Health-led FRIENDS vs usual school provision: GBP 18/RCADS score, GBP 14,617/QALY with < 35% probability of CE at any WTP threshold for a QALY. Health-led FRIENDS vs school-led FRIENDS; dominated (ICER of GBP 0/RCADS score, GBP -3/QALY). Authors' conclusion: not cost-effective
15	Uegaki (2011), Netherlands	Trial based cost utility analysis	Societal	NA/NA	Cost related to the health care sector, other sector and patient/family resource use and productivity loss: 2006 EUR	QALY	Dominated with 20% probability of CE at a WTP of EUR 0 through EUR 50,000/QALY. Authors' conclusion: not cost-effective
16	Underwood (2013), UK	Trial based cost utility analysis	Healthcare sector	NA/NA	Intervention and health-care cost: 2010 GBP	QALY	Dominated with 33% probability of CE at a WTP of GBP 20,000/QALY and 37% at GBP 30,000/QALY Authors' conclusion: not cost-effective
17	van den Berg (2011), Netherlands	Model based cost utility analysis	Societal	4.0% for costs and 1.5% for effects/5 years	Intervention, healthcare and societal cost: 2008 EUR	DALY	ICER from health care perspective: EUR 1400 /DALY with 79% probability of CE at a WTP of EUR 20,000/DALY ICER from societal perspective: Cost saving with 83% probability of CE at a WTP of EUR 20,000/DALY. Authors' conclusion: cost-effective
18	Ahern et al. (2018), Europe	Trial based cost utility analysis	Payer	NA/NA	Direct costs (intervention and travel): 2010 EUR	Incident suicide attempt, incident severe suicidal ideation; QALY	QPR for suicidal attempt: EUR 90.43/1% point reduction in incident and EUR 120,567/QALY. QPR for severe suicidal ideation: Dominated. Authors' conclusion: not cost-effective
							YAM for suicidal attempt: EUR 34.83/1% point reduction in incident and EUR 47,017/QALY YAM for severe suicidal ideation: EUR 45.42/1% point reduction in incident and EUR 48,216/QALY and at a WTP of EUR 52,000 per gained QALY and greater, it had a 44% probability of CE in preventing a suicide attempt. For severe suicidal ideation, YAM had the greatest probability of being cost-effective 45% at a WTP of EUR 50,000 and greater. Authors' conclusion: cost-effective
19	Comans (2013), Australia	Model based cost utility analysis	Societal	5.0%/1 year	Intervention, healthcare and productivity costs: 2011, AUD	QALY	ProfScreen for suicidal attempt: EUR 52.29/1% point reduction in incident and EUR 64,050/QALY ProfScreen for severe suicidal ideation: EUR 102.48/1% point reduction in incident and EUR 108,790/QALY. Authors' conclusion: not cost-effective. Dominant. Authors' conclusion: cost-effective

*AUD* Australian dollar, *CE* cost-effectiveness, *CES-D* Centre for Epidemiologic Studies Depression Scale, *DFDs* depression free days, *DFYs* depression-free years, *EUR* euro, *GBP* Great Britain pound, *HADS-A* Hospital Anxiety and Depression Scale, *NZD* New Zealand dollar, *QPR* question, persuade, refer, *RCADS* Revised Child Anxiety and Depression Scale, *RTW* return to work, *SEK* Swedish krona, *SMFQ* Short Mood and Feelings Questionnaire, *USD* United States dollar, *VAS* Visual Analogue Scale, *WTP* willingness to pay, *YAM* Youth Aware of Mental Health programme

^*^Dominated: an intervention is dominated if it costs more and is less effective than the comparator, therefore NOT cost-effective

^**^Dominant: An intervention is dominant if it costs less and is more effective than the comparator, therefore cost-effective

### Population, Age Groups, Type of Intervention and Arena

Mental health interventions targeted different population groups. Seven studies (Buntrock et al. 2017; Fernandez et al. 2018; Ophuis et al. 2018; Ride et al. 2016; Uegaki et al. 2011; van den Berg et al. 2011; van Oostrom et al. 2010) evaluated interventions delivered to the general population, mainly adults over 18 years old. Four interventions were delivered at primary and maternity care centers (Fernandez et al. 2018; Ophuis et al. 2018; Ride et al. 2016; van den Berg et al. 2011), two were delivered in the workplace (Uegaki et al. 2011; van Oostrom et al. 2010) and one was an internet-based cognitive behavioral therapy (CBT) intervention (Buntrock et al. 2017). Five of these interventions were cost-effective (Buntrock et al. 2017; Fernandez et al. 2018; Ophuis et al. 2018; Ride et al. 2016; van den Berg et al. 2011). Four studies (Bosmans et al. 2014; Coulton et al. 2015; Lewis et al. 2017; Underwood et al. 2013) evaluated interventions specifically aimed at older people over the age of 60, with only two of these being cost-effective (Coulton et al. 2015; Lewis et al. 2017). Six studies (Anderson et al. 2014; Lee et al. 2017; Lynch et al. 2005; Mihalopoulos et al. 2012; Philipsson et al. 2013; Stallard et al. 2015) evaluated seven interventions targeting children and adolescents under 18 years. The majority of these interventions were delivered in school settings, and all except one (Anderson et al. 2014) were cost-effective. In general, most of the interventions were provided in collaboration between different societal sectors, such as health care, elderly care, educational sector and labor market.

Of the two suicide prevention evaluations, one study (Ahern et al. 2018) analyzed three different universal interventions at school, for school pupils, one of which was cost-effective. The second study (Comans et al. 2013) evaluated an indicated intervention given to people bereaved by suicide, and was cost-effective.

### Quality and Transferability to the Swedish Context

Four studies (Anderson et al. 2014; Buntrock et al. 2017; Lewis et al. 2017; van Oostrom et al. 2010) aimed to promote mental health were considered to be of high quality, but none of the studies that evaluated suicide preventive interventions were considered high quality. Of the high-quality studies, all evaluated interventions were indicated. The interventions were mainly CBT based and delivered in the community and workplace for sick employees to return to work. Two studies adopted a societal perspective (Buntrock et al. 2017; van Oostrom et al. 2010), the other used a health and social care sector perspective (Anderson et al. 2014; Lewis et al. 2017). Two interventions (Buntrock et al. 2017; Lewis et al. 2017) were cost-effective.

Results of the nine studies (around 50% of included articles) were considered to have high potential of transferability to the Swedish context (Ahern et al. 2018; Anderson et al. 2014; Bosmans et al. 2014; Buntrock et al. 2017; Lewis et al. 2017; Philipsson et al. 2013; Uegaki et al. 2011; van den Berg et al. 2011; van Oostrom et al. 2010). Only two studies evaluated interventions delivered to Swedish populations (Philipsson et al. 2013; Ahern et al. 2018). The first evaluated a dance intervention for teenage girls with mental health problems and was cost-effective from a societal perspective. The second study (Ahern et al. 2018) evaluated three suicide preventive interventions, where only one was cost-effective according to the Swedish willingness-to-pay threshold.

## Discussion

### Main Findings

This review aimed to contribute to the literature supporting the cost-effectiveness of preventive interventions targeting mental health and suicide in public health. Our findings demonstrated that a number of public health interventions aiming to improve mental health and prevent suicidal thoughts and behaviour were cost-effective and certainly need to be considered in any package of public health initiatives targeting health promotion and disease prevention. Most of the studies showing cost-effective results were empirical evaluations, which means that the knowledge regarding the long-term economic effects of the interventions was limited due to short time horizons. A majority of the interventions were indicated, such as CBT based psychological interventions, and most focused on depression and anxiety prevention. Only two studies evaluated four preventive interventions for suicide, with two of them being cost-effective. About half of the included studies had high potential for transferability of results to the Swedish context.

There is a strong economic evidence base regarding universal and indicated school-based psychological interventions aimed to improve the mental health of children and adolescents. There is also a compelling economic case for investing in actions in the workplace, as well as in preventive strategies in primary and maternity care. Evidence has likewise shown that indicated actions targeted at groups of older people who are at high risk for depression could be cost-effective. Suicide is one of the most well-known potential consequences of poor mental health, but the evidence on cost-effective actions to prevent suicide is limited, in spite of numerous effective suicide prevention interventions (Zalsman et al. 2016).

### Challenges in Using Economic Evidence

Economic evaluation has two defining features; the first is that both costs and consequences (or benefits) of alternative interventions are considered; the second is that choices between different interventions must be made (Drummond et al. 2005). Choices are often based on many reasons, which may or may not be obvious. Therefore, economic evaluation can provide decision-makers with information regarding the economic value of interventions, and assist with the difficult decision of resource allocation.

This systematic review aimed to improve decision making by providing knowledge to public health professionals on the current economic evidence regarding interventions to improve mental health and prevent suicide. While the number of studies identified in this area remains limited, their cost-effectiveness results may favorably compete with the cost-effectiveness of other public health interventions, and thus support investment in prevention, promotion and early interventions in mental health. However, attention toward such interventions is still insufficient (Arango et al. 2018). As it was highlighted by McDaid et al (2019), promoting and protecting mental health usually involves many societal sectors, more than solely health services. Collaboration between different sectors such as educational, the labor market and elderly care is necessary but can be problematic, because improvement of mental health is not often a primary objective for these sectors. Our results confirm that interventions given in collaboration between different societal sectors were cost-effective, which should encourage decision makers to commit resources to mental health interventions.

This review included both empirical and model-based economic evaluations, which are different types of evaluation frameworks that are often not comparable in terms of follow-up time, range of comparators and case-mix of patients (Anderson 2010). Empirical studies evaluate actual costs and health consequences, though usually in the short- term, while model-based studies estimate future potential costs and consequences. Since the purpose was to create a general picture of the state of knowledge and not to compare individual studies, this review provides a broad view of the evidence regarding both the short-(empirical) and long-term (model-based) cost-effectiveness of preventive interventions. In our opinion, that may be important for decision-makers to choose appropriate interventions according to specific goals and accounting for different time horizons.

### Generalizability of Findings

One of the aims of this study was to assess the transferability of the results to the Swedish context. Given the limited number of relevant studies, it is important to acknowledge which results of the review may be used within a certain context, in this case Sweden. Due to the specific context of many interventions, the level of generalizability and transferability may be restricted. That is why different expert panels on methodological considerations for health economic evaluation (Tordrup et al. 2017) emphasize the importance of detailed reporting on all variables included in the economic evaluation to assess the transferability to different country settings. Considering those, we chose national checklists developed by the Swedish Agency for Health Technology Assessment, which incorporated a transferability assessment section as part of quality assessment. The main reason was that most of the available checklists (Walker et al. 2012) were not created to be used as tools to assess the relation between study components and the setting of potential decision-makers. In this study, transferability was presented as part of the general results, which may benefit decision-makers. Our analyses have shown that the cost-effectiveness results of nine studies have a high potential of transferability to the Swedish context, hence promoting investment in those interventions.

### Limitations

The limited number of studies included in this review may be a reflection of the difficulty in conducting economic evaluations in the areas of mental health and suicide prevention, as mentioned previously (Rush et al. 2004). Our exclusion criteria and review protocol also excluded studies evaluating selective interventions due to generalizability issues. In addition, studies were excluded if they had not used a generic preference-based outcome measure (QALY or DALY). Therefore, relevant and high quality cost-effectiveness studies using clinical outcomes that could support decision-making in specific clinical context may have been missed. However, it is methodologically more difficult to generalize results of economic evaluations reporting costs per specific clinical measure, since the threshold for cost-effectiveness is based on the willingness to pay for a QALY/DALY. For the same reason we also excluded cost–benefit analyses (CBAs), which might have shown favourable results in some areas, such as workplace interventions where CBAs are commonly used. Similarly, limiting the geographical scope may have led to a lower number of hits, but as most of articles outside the included countries would be less transferable to the Swedish context, it is unlikely that relevant studies were missed.

In the most studies adding productivity losses was used to claim societal perspective, which is relevant for the working population. Contrary to that, societal perspective for children may include educational and/or social services sector costs. This also, amongst other things mentioned, makes it difficult to compare across studies. In this regard, readers should consider the target population in each study and interpret results in light of this.

## Conclusion

Public health interventions to improve mental health have a high potential to yield considerable economic benefits to society, however, evidence on the cost-effectiveness of suicide prevention is limited. Most economic evaluations of interventions for the prevention of mental health problems were cost-effective, with about half had a high potential for transferability of results to the Swedish setting.

## Electronic supplementary material

Below is the link to the electronic supplementary material.

Supplementary file1 (DOCX 23 kb)
