# Supplementary material for: Economic Evaluations of Public Health Interventions to Improve Mental Health and Prevent Suicidal Thoughts and Behaviours: A Systematic Literature Review
Source: Adm Policy Ment Health. 2020 Jul 30;48(2):299–315. doi: 10.1007/s10488-020-01072-9 (PMC7870636; doi:10.1007/s10488-020-01072-9)
Supplement: Supplementary file 1 — Supplementary file1 (DOCX 23 kb) [file 10488_2020_1072_MOESM1_ESM.docx]

**Supplementary material**

Appendix 1. Search strategy

PubMed via NLM, date searched: 20181121,

**Limits**; English, publication year 2000-2018, full text available

|  | Search term | hits |
| --- | --- | --- |
|  | **Intervention** |  |
| 1 | “Health promotion/Methods” [MeSH] OR “Early intervention (Education)” [MeSH] OR “Public health” [MeSH:NoExp] OR “Health education” [MeSH:NoExp] OR “Preventive Health Services” [MeSH:NoExp] OR “Primary Prevention” [MeSH:NoExp] OR “Preventive medicine” [MeSH:NoExp] OR “prevention and control” [SH] OR “health behavior” [MeSH] OR “health risk behavior” [MeSH] OR “risk reduction behavior” [MeSH] OR “life style” [MeSH]) OR control [tiab] OR prevent* [tiab] OR healthy liv* [tiab] OR healthy weight [tiab] OR behavior chang*[tiab] OR behavior modif*[tiab] OR behavior program*[tiab] OR behavior therap* [tiab] OR behavioral chang* [tiab] OR behaviour chang*[tiab] OR behaviour modif*[tiab] OR behaviour program*[tiab] OR behaviour therap*[tiab] OR behavioural chang*[tiab] OR cessation [tiab] OR health campaign* [tiab] OR well* program [tiab] OR public health [tiab] OR (public health [tiab] AND (intervent* [ti] OR strateg* [tiab] OR program* [tiab])) | 5706101 |
|  | **Study design** |  |
| 2 | Costs and Cost Analysis [MeSH] OR “Economic evaluation*” [tiab] OR Cost* [tiab] | 406193 |
|  | **Outcome** |  |
| 3 | “Quality-Adjusted Life Years” [MeSH] OR QALY* [tiab] OR “disability-adjusted life year*”[tiab] OR DALY* [tiab] OR adjusted life year* [tiab] | 15880 |
|  | **Physical activity** |  |
| 4 | (“Physical Fitness” [MeSH] OR “sports” [MeSH] OR “Exercise” [MeSH] OR “Leisure Activities” [MeSH] OR “sedentary lifestyle” [MeSH] OR “Healthy lifestyle” [MeSH] OR Exercise* [tiab] OR physical activit* [tiab] OR physical exercise* [tiab] OR acute exercise* [tiab] OR isometric exercise* [tiab] OR training exercise* [tiab] OR aerobic exercise* [tiab] OR leisure* [tiab] OR leisure activit* [tiab] OR sport* [tiab] OR athletic* [tiab] OR walk* [tiab] OR bik* [tiab] OR fitness [tiab] OR “weight loss” [tiab] OR sedentar* [tiab] OR Aerobic* [tiab] OR athletic* [tiab]) NOT (rehab* [ti] OR physiotherap*[ti]) | 485900 |
|  | **Combined** |  |
| 5 | #1 AND #2 AND #3 AND #4 | **531** |
|  | **Diet** |  |
| 6 | Diet [Majr] OR Feeding behavior [MeSH] OR eating [MeSH] OR obesity [MeSH] OR Healthy diet [MeSH] OR overweight [MeSH] OR fast foods [MeSH] OR Body Mass Index [MeSH] OR unhealthy diet [tiab] OR healthy diet* [tiab] OR Behavior, Feed* [tiab] OR Body Mass Index [tiab] OR BMI [tiab] OR Feeding Behavior* [tiab] OR eating behavior* [tiab] OR feeding pattern* [tiab] OR food habit* [tiab] OR eating habit* [tiab] OR dietary habit* [tiab] OR unhealthy eat* [tiab] OR excess eat* [tiab] OR food intake [tiab] OR ingestion [tiab] OR obes* [tiab] OR overweigh* [tiab] OR fast food* [tiab] OR Convenience Food* [tiab] OR Ready-Prepared Food* [tiab] OR Ready-To-Eat Meal* [tiab] OR junk food* [tiab] OR food choice* [tiab] OR food preference* [tiab] | 513557 |
|  | **Combined** |  |
| 7 | #1 AND #2 AND #3 AND #6 | **423** |

Abstracts on the *combined* raw are to be extracted and included in the screening

[MeSH] = Term from the Medline controlled vocabulary, including terms found below this term in the MeSH hierarchy

[SH] = Subject heading

[MeSH:NoExp] = Does not include terms found below this term in the MeSH hierarchy

[tiab]= title and abstract

*= Truncation

**EEs of Prevention and promotion public health interventions**

PsycINFO via EBSCO, Date searched: 20181122, Limits; English, publication year 2000-2018, peer reviewed

|  | Search term | | | hits |
| --- | --- | --- | --- | --- |
|  | **intervention** | | |  |
| 1 | DE “Health promotion” OR DE “intervention” OR DE “Early intervention” OR DE “Public health” OR DE “Public health services” OR DE “Health education” OR DE “Prevention” OR DE “Preventive medicine” OR DE “health behavior” OR DE “behavior change” OR DE “behavior modification” OR DE “Lifestyle Changes” OR TI (health promotion OR health risk behavior* OR risk reduction behavior* OR life style* OR control OR prevent* OR healthy liv* OR healthy weight* OR behavior chang* OR behavior modif* OR behavior program* OR behavior therap* OR behavioral chang* OR behaviour chang* OR behaviour modif* OR behaviour program* OR behaviour therap* OR behavioural chang* OR cessation* OR health campaign* OR well* program OR public health OR intervent*) OR AB (health promotion OR health risk behavior* OR risk reduction behavior* OR life style* OR control OR prevent* OR healthy liv* OR healthy weight* OR behavior chang* OR behavior modif* OR behavior program* OR behavior therap* OR behavioral chang* OR behaviour chang* OR behaviour modif* OR behaviour program* OR behaviour therap* OR behavioural chang* OR cessation* OR health campaign* OR well* program OR public health OR intervent*) | | | 674,802 |
|  | Study design | | |  |
| 2 | DE “Costs and Cost Analysis” OR TI (“Economic evaluation*” OR “Cost*”) OR AB (“Economic evaluation*” OR “Cost*”) | | | 62,995 |
|  | **Outcome** | | |  |
| 3 | TI (“Quality-Adjusted Life Year*” or “QALY*” OR “disability-adjusted life year*” OR DALY* OR adjusted life year*) OR AB (“Quality-Adjusted Life Year*” or “QALY*” OR “disability-adjusted life year*” OR DALY* OR adjusted life year*) | | | 3,436 |
|  | | **Physical activity** |  | |
| 4 | | DE “Physical Activity” OR DE “Physical Fitness” OR DE “Sports” OR DE “Exercise” OR DE “Aerobic Exercise” OR DE “lifestyle” OR DE “Active Living” OR DE “Physical Mobility” OR DE “Leisure Time” OR  DE “Sedentary Behavior” OR DE “weight control” OR DE “weight loss” OR TI (“Leisure Activit*” OR Exercise* OR physical activiti* OR physical exercise* OR acute exercise* OR isometric exercise* OR training exercise* OR aerobic exercise* OR leisure* OR leisure activit* OR sport* OR athletic* OR walk* OR bik* OR fitness* OR “sedentary lifestyle” OR “weight loss” OR sedentar* OR Aerobic* OR athletic*) OR AB (“Leisure Activit*” OR Exercise* OR physical activiti* OR physical exercise* OR acute exercise* OR isometric exercise* OR training exercise* OR aerobic exercise* OR leisure* OR leisure activit* OR sport* OR athletic* OR walk* OR bik* OR fitness* OR “sedentary lifestyle” OR “weight loss” OR sedentar* OR Aerobic* OR athletic*) | 105,463 | |
|  | | **Combined** |  | |
| 5 | | #1 AND #2 AND #3 AND #4 | **69** | |
|  | | **Diet** |  | |
| 6 | | DE “Diets” OR DE “Eating behavior” OR DE “Obesity” OR DE “Overweight” OR DE “Body Mass Index” OR DE “Weight Gain” OR DE “Food Preferences” OR TI (feeding behavior* OR eat* OR obes* OR overweight OR fast food* OR unhealthy diet OR healthy diet OR Behavior, Feed* OR Feeding Behavior* OR eating behavior* OR feeding pattern* OR food habit* OR eating habit* OR dietary habit* OR unhealthy eat* OR excess eat* OR food intake OR ingestion OR overweight OR fast food* OR Convenience Food* OR Ready-Prepared Food* OR Ready-To-Eat Meal* OR junk food* OR food choice* OR food preference* OR BMI) OR AB unhealthy diet OR healthy diet OR Behavior, Feed* OR Feeding Behavior* OR eating behavior* OR feeding pattern* OR food habit* OR eating habit* OR dietary habit* OR unhealthy eat* OR excess eat* OR food intake OR ingestion OR overweight OR fast food* OR Convenience Food* OR Ready-Prepared Food* OR Ready-To-Eat Meal* OR junk food* OR food choice* OR food preference* OR BMI) | 95,044 | |
|  | | **Combined** |  | |
| 7 | | #1 AND #2 AND #3 AND #6 | **54** | |

Abstracts on the *combined* raw are to be extracted and included in the screening

AB = Abstract

DE = Term from the thesaurus

MM = Major Concept

TI = Title

ZC = Methodology Index

* = Truncation

“ ” = Citation Marks; searches for an exact phrase

**EEs of Prevention and promotion public health interventions**

Web of Science Core collection, Date searched: 20181123,

**Limits**: Document type; article, Language; English, publication year; 2000-2018

|  | Search term | hits |
| --- | --- | --- |
|  | **intervention** |  |
| 1 | TS=(((intervention OR service OR program* OR strategy) AND “public health”) OR “Early intervention” OR “Health promotion” OR “Health education” OR Prevent* OR “health behavio$r” OR lifestyle OR “health risk behavio$r” OR “risk reduction behavior$r” OR “healthy liv*” OR “healthy weight” OR (behavio$r* AND (chang* OR modif* OR program* OR therap* OR health)) OR cessation OR “health campaign*” OR “wellness program*”) | 1,667,453 |
|  | **Study design** |  |
| 2 | TS=(“Economic evaluation*” OR “Cost*”) | 814,869 |
|  | **Outcome** |  |
| 3 | TS= (“Quality-Adjusted Life Year*” or “QALY*” OR “disability-adjusted life year*” OR DALY* OR “adjusted life year*”) | 12,319 |
|  | **Physical activity** |  |
| 4 | TS= ((“Physical activit*” OR “Physical Fitness” OR exercis* OR sport* OR walk* OR bik* OR “weight loss” OR physical NEAR/1 train* OR “Active Liv*” OR Physical NEAR/1 Mobilit* OR “weight control*” OR “weight loss” OR “Leisure Activit*” OR “physical exercise*” OR fitness* OR sedentar* OR Aerobic* OR athletic*) NOT (rehab* OR physiotherap*)) | 593,065 |
|  | **Combined** |  |
| 5 | #1 AND #2 AND #3 AND #4 | **221** |
|  | **Diet** |  |
| 6 | TS=(“Unhealthy diet*” OR “healthy diet*” OR “eating habit*” OR ((Eat* OR feed* OR food) AND behavio$r) OR “fast food*” OR “carbonated drink*” OR obes* OR overweight OR “junk food” OR “excess eat*” OR “food preference” OR “food choice*” OR “eating practice*” OR “feeding pattern*” OR “Convenience Food*” OR “Ready-Prepared Food*” OR “Ready-To-Eat Meal*” OR “food consum*” OR “Body Mass Index” OR BMI OR “feeding habit*” OR “dietary habit*” OR “unhealthy eat*” OR “healthy eat*”) | 468,144 |
|  | **Combined** |  |
| 7 | #1 AND #2 AND #3 AND #6 | **180** |

Abstracts on the *combined* raw are to be extracted and included in the screening

TS = Topic (searches title, abstract and keywords)

TI= title

* = Truncation

“ ” = Citation Marks; searches for an exact phrase

**EEs of Prevention and promotion public health interventions**

NHS EED and HTA, Date searched: 20181127,

**Limits**: publication year; 2000-2018

|  | Search term | Hits (NHSEED/HTA) | |
| --- | --- | --- | --- |
|  | **intervention** |  | |
| 1 | (MeSH DESCRIPTOR preventive health services EXPLODE ALL TREES) OR (MeSH DESCRIPTOR Public health) OR (MeSH DESCRIPTOR Preventive Medicine) OR (MeSH DESCRIPTOR health behavior EXPLODE ALL TREES) OR (MeSH DESCRIPTOR risk reduction behavior) OR (MeSH DESCRIPTOR life style EXPLODE ALL TREES) OR Early NEAR3 intervention*:TI OR Health education* OR Prevent*:TI OR public health:TI OR healthy liv* OR healthy NEAR weigh* OR behavior chang* OR behavior modif* OR behavior therap* OR behavioral chang* OR behaviour chang* OR behaviour modif* OR behaviour therap* OR behavioural chang* OR cessation OR health campaign* OR wellness program* OR (public health AND (intervent* OR program* OR strateg*)) | 4458/  1629 | |
|  | **Study design** |  | |
| 2 | ((MeSH DESCRIPTOR costs and cost analysis EXPLODE ALL TREES) OR Economic evaluation* OR Cost*) | 14762/ 3317 | |
|  | **Outcome** |  | |
| 3 | (MeSH DESCRIPTOR Quality-Adjusted Life Years EXPLODE ALL TREES) OR QALY* OR Quality-Adjusted Life Year* OR disability-adjusted life year* OR DALY* OR adjusted life year* | 4607/  338 | |
|  | **Physical activity** | |  |
| 4 | (MeSH DESCRIPTOR Exercise EXPLODE ALL TREES) OR (MeSH DESCRIPTOR Leisure Activities) OR (MeSH DESCRIPTOR Sedentary Lifestyle) OR (MeSH DESCRIPTOR Healthy Lifestyle) OR (MeSH DESCRIPTOR Physical Fitness) OR (MeSH DESCRIPTOR Sports) OR (MeSH DESCRIPTOR weight loss) OR Exercise* OR physical activit* OR physical train* OR leisure* OR sport* OR athletic* OR walk* OR bik* OR fitness OR weight loss OR sedentar* OR Aerobic* | | 2976/  653 |
|  | **Combined** | |  |
| 5 | #1 AND #2 AND #3 AND #4 | | **553/6** |
|  | **Diet** | |  |
| 6 | (MeSH DESCRIPTOR weight Gain) OR (MeSH DESCRIPTOR Healthy Diet) OR (MeSH DESCRIPTOR Feeding Behavior) OR (MeSH DESCRIPTOR Food Preferences) OR (MeSH DESCRIPTOR Fast Foods) OR (MeSH DESCRIPTOR Eating) OR (MeSH DESCRIPTOR Obesity) OR (MeSH DESCRIPTOR Body Mass Index) OR (MeSH DESCRIPTOR Body Size EXPLODE ALL TREES) OR (MeSH DESCRIPTOR overweight) OR Unhealthy diet* OR healthy diet* OR eating habit* OR ((Eat* OR feed* OR food) AND behavior) OR fast food* OR carbonated drink* OR obes* OR overweight OR junk food* OR excess eat* OR food preference* OR food choice* OR eating practice* OR feeding pattern* OR Convenience Food* OR Ready-Prepared Food* OR Ready-To-Eat Meal* OR food consum* OR Body Mass Index OR BMI OR feeding habit* OR dietary habit* OR unhealthy eat* OR healthy eat* | | 482/307 |
|  | **Combined** | |  |
| 7 | #1 AND #2 AND #3 AND #6 | | **87/3** |

Abstracts on the *combined* raw are to be extracted and included in the screening

[MeSH] = Medical subject heading, including terms found below this term in the MeSH hierarchy

TI= title

* = Truncation

“” = Citation Marks; searches for an exact phrase
